# Neutrophil-to-Lymphocyte Ratio as a Predictor of In-Hospital Mortality and Complications in Acute Coronary Syndrome: A Cross-Sectional Study

**DOI:** 10.7759/cureus.106745

**Published:** 2026-04-09

**Authors:** Anusuri Priyanka, Namagiri Sri Ramaraju, Vaka Anuhya Vidya Devi, Suthanthira Kannan, Kanaka Mahalakshmi Sai Lahari

**Affiliations:** 1 Internal Medicine, GSL Medical College and General Hospital, Rajahmundry, IND; 2 Cardiology, KIMS Hospitals, Rajahmundry, IND; 3 Internal Medicine, Sree Latha Hospitals, Rajahmundry, IND; 4 Community Medicine, Employee's State Insurance Corporation (ESIC) Medical College and Hospital, Chennai, IND

**Keywords:** : acute coronary syndrome, neutrophil to lymphocyte ratio (nlr), patient-centered outcomes research, risk predictors, south india population

## Abstract

Background

Acute coronary syndrome (ACS) is a major cause of morbidity and mortality worldwide. Inflammation plays a key role in its pathogenesis, and the neutrophil-to-lymphocyte ratio (NLR) has emerged as a potential prognostic marker. This study aimed to evaluate its role in predicting in-hospital complications and mortality among patients admitted with ACS.

Methods

This hospital-based cross-sectional observational study included 103 patients admitted with ACS. Patients were categorized into two groups based on NLR: <7 (n = 57, 55.3%) and ≥7 (n = 46, 44.7%). Clinical parameters, laboratory findings, and outcomes, including complications, heart failure, arrhythmias, and mortality, were recorded and analyzed.

Results

Among the study participants, 76 (73.7%) were male and 27 (26.3%) were female, with the majority aged 40-60 years. Hypertension was present in 42 (40.7%) patients, and diabetes mellitus was present in 57 (55.4%) patients. Elevated NLR (≥7) was significantly associated with left ventricular dysfunction (38/63, 60.3%), compared to 8/40 (20.0%) in the low-NLR group (p < 0.001). Heart failure was more frequent in the high-NLR group (34/54, 63.0%) than in the low-NLR group (20/54, 37.0%) (p < 0.001). Arrhythmias were observed in 24/36 (66.7%) patients with high NLR, compared to 12/36 (33.3%) patients with low NLR (p = 0.001). Complications were significantly more common in patients with elevated NLR (43/46, 93.5%) than in those with lower NLR (40/57, 70.2%) (p = 0.003). Mortality was also significantly higher in the high-NLR group (21/27, 77.8%) than in the low-NLR group (6/27, 22.2%) (p < 0.001).

Conclusion

Elevated NLR is a strong predictor of adverse outcomes in ACS. It can serve as a simple and effective tool for early risk stratification and prognostication in clinical practice.

## Introduction

Cardiovascular diseases (CVDs) remain the leading cause of mortality worldwide, accounting for a substantial proportion of global deaths. Among these, coronary artery disease, particularly acute coronary syndromes (ACS), represents a major contributor to fatal outcomes such as myocardial infarction and heart failure [1]. Ischemic heart disease continues to be the single most significant factor driving the increasing global burden of CVD, with ACS being one of the most common causes of hospital admission among affected patients.

ACS encompasses a spectrum of clinical conditions characterized by acute myocardial ischemia. This includes unstable angina (UA), non-ST-segment elevation myocardial infarction (NSTEMI), and ST-segment elevation myocardial infarction (STEMI). The diagnosis of ACS requires the integration of clinical history, ECG findings, and biochemical markers. However, in the early hours following presentation, differentiating between these entities can be challenging, as biomarkers of myocardial necrosis may initially remain within normal limits [2]. This diagnostic uncertainty highlights the need for additional markers that can aid in early risk stratification and prognostication.

Inflammation plays a central role in the pathogenesis of atherosclerosis and its complications. Early atherosclerotic lesions are dominated by immune cells, whose effector molecules contribute to plaque progression and instability. Activation of inflammatory pathways is a key event leading to plaque rupture and subsequent thrombus formation, ultimately resulting in ACS [3]. Among the various inflammatory cells, neutrophils have been implicated in mediating plaque destabilization through the release of proteolytic enzymes and reactive oxygen species. These processes contribute to endothelial injury, activation of coagulation pathways, microvascular obstruction, and myocardial necrosis, largely driven by the release of proinflammatory cytokines [4].

In addition to neutrophilia, ACS is also associated with lymphopenia, which is thought to occur because of physiological stress and activation of the neurohormonal axis. The resultant increase in cortisol levels induces apoptosis of lymphocytes, thereby reducing their circulating numbers [5]. Consequently, the neutrophil-to-lymphocyte ratio (NLR) emerges as a composite marker that reflects both the innate inflammatory response (via neutrophils) and the adaptive immune response (via lymphocytes). This dual representation makes NLR a potentially valuable surrogate marker of systemic inflammation in patients with ACS [6].

Leukocytosis is commonly observed in patients presenting with ACS and has been shown to correlate with short-term morbidity and mortality [7]. The NLR, in particular, represents a dynamic balance between neutrophil-mediated inflammatory activity and lymphocyte-mediated regulatory mechanisms. Elevated NLR has been associated with increased systemic inflammation and may serve as an indicator of adverse cardiovascular outcomes [8]. Furthermore, NLR may also reflect myocardial remodeling processes following ischemia-reperfusion injury, thereby providing additional prognostic value [9].

Among various hematological indices, NLR has demonstrated superior predictive ability in identifying high-risk patients for myocardial infarction and mortality [10]. It has also been shown to predict long-term outcomes in patients with ST-elevation myocardial infarction and in those undergoing percutaneous coronary intervention (PCI) [11]. Although cardiac biomarkers such as Troponin-I are well-established indicators for diagnosing acute myocardial infarction, their prognostic utility may be limited in certain clinical conditions. For instance, elevated troponin levels are often observed in patients with renal insufficiency, in whom it may be difficult to distinguish between ischemic injury and chronic myocardial damage [12]. In such scenarios, NLR may provide additional diagnostic and prognostic value in identifying true ischemic events [13,14].

Neutrophils are considered markers of active inflammation, while lymphocytes reflect regulatory and protective pathways within the immune system. The NLR, calculated by dividing the absolute neutrophil count by the absolute lymphocyte count, serves as a simple, cost-effective, and readily available marker of systemic inflammation. Given the growing evidence supporting the prognostic significance of NLR, this study aimed to evaluate its role in predicting in-hospital complications and mortality among patients admitted with acute coronary syndrome. By identifying high-risk patients early in the disease course, NLR may serve as a valuable tool to guide clinical decision-making and improve patient outcomes.

## Materials and methods

Study design and setting

This hospital-based cross-sectional observational study was conducted in the Cardiology Division of the Department of Medicine at GSL Medical College and General Hospital. The study aimed to evaluate the role of the NLR in predicting morbidity and mortality among patients presenting with ACS. The hospital is a tertiary care center that caters to a large number of patients with cardiovascular diseases, providing an appropriate setting for the study of acute cardiac conditions.

Study period

The study was carried out over a period of approximately 20 months, from October 1, 2018, to June 1, 2020. During this period, all eligible patients admitted with a diagnosis of ACS were screened for inclusion in the study.

Study population and sample size

The study population comprised patients admitted to the cardiology division with a diagnosis of ACS. Based on previous literature [10], a sample size of 100 was calculated using NMaster software. All consecutive eligible patients admitted with ACS between October 1, 2018, and June 1, 2020, were enrolled. A total of 103 patients fulfilled the inclusion criteria and provided written informed consent. All consecutive eligible patients during the study period were enrolled, ensuring that the sample represented the spectrum of ACS cases encountered in routine clinical practice.

Inclusion and exclusion criteria

Patients presenting with acute chest pain and subsequently diagnosed with ACS were included in the study, provided they gave informed consent to participate. The diagnosis of ACS was based on clinical presentation, electrocardiographic findings, and cardiac biomarkers. Patients were excluded if they had conditions that could independently alter leukocyte counts or inflammatory status. These included individuals with fever due to active infection, known hematological disorders, underlying liver disease, connective tissue disorders, or malignancy. Patients receiving statin therapy were also excluded, as statins may influence inflammatory markers, including NLR. These criteria were applied to minimize confounding factors and ensure accurate assessment of the relationship between NLR and clinical outcomes in ACS.

Clinical evaluation

All study participants underwent a detailed clinical evaluation using a structured proforma. This included the recording of demographic details, presenting complaints, past medical history, and risk factors such as hypertension and diabetes mellitus. A thorough general physical examination and systemic examination were performed in all patients. Particular attention was given to identifying complications associated with ACS, including heart failure, pulmonary edema, hypotension, arrhythmias, and other adverse events. Patients were also followed during their hospital stay to assess clinical outcomes, including mortality.

Measurement of clinical and biochemical parameters

Blood Pressure Assessment

Blood pressure was measured in all patients in the supine position using a standard sphygmomanometer. A reading of ≥140/90 mmHg was considered indicative of hypertension according to the American Heart Association (AHA) guidelines [15]. Patients who were already on antihypertensive medications were also categorized as hypertensive irrespective of their current blood pressure readings.

Blood Glucose Estimation

Random blood sugar (RBS) levels were measured for all participants. An RBS value of >200 mg/dL was considered diagnostic of diabetes mellitus as per the American Diabetes Association (ADA) guidelines [16]. Patients with a known history of diabetes or those on antidiabetic medications were also classified as diabetic.

Renal Function Tests

Blood urea and serum creatinine levels were measured to assess renal function. Serum creatinine values greater than 1.2 mg/dL and blood urea levels exceeding 40 mg/dL, particularly when associated with decreased urine output, were considered indicative of renal dysfunction or possible acute kidney injury.

Lipid Profile

A lipid profile was obtained for all patients, including serum triglyceride and total cholesterol levels. Serum triglyceride levels greater than 150 mg/dL and total cholesterol levels exceeding 200 mg/dL were considered abnormal, indicating hyperlipidemia.

Electrocardiographic and imaging evaluation

12-Lead ECG

All patients underwent a standard 12-lead ECG at admission using a Mindray BeneHeart R12 machine. The ECG was used to classify the type of ACS, including anterior wall myocardial infarction, inferior wall myocardial infarction, anterolateral infarction, septal infarction, and other patterns. The ECG was also evaluated for the presence of arrhythmias such as atrial fibrillation, heart blocks, bundle branch blocks, and ventricular tachycardia.

2D Echocardiography

All participants underwent two-dimensional echocardiography (2D echo) using a Philips HD11XE system to assess cardiac function. Left ventricular ejection fraction (LVEF) was measured, with values >50% considered normal and values <50% considered indicative of left ventricular dysfunction.

NLR

Complete blood counts were performed for all patients using an automated hematology analyzer (Erba Mannheim H560). Parameters obtained included total white blood cell count, differential count, absolute neutrophil count, and absolute lymphocyte count. The NLR was calculated by dividing the absolute neutrophil count by the absolute lymphocyte count. NLR was measured at the time of admission and analyzed in relation to clinical outcomes, particularly the development of complications and in-hospital mortality. An NLR value of ≥7 was considered elevated and was evaluated as a predictor of adverse outcomes.

The NLR was calculated as the absolute neutrophil count divided by the absolute lymphocyte count. For categorical analysis, patients were grouped as NLR <7 and NLR ≥7. The cut-off of 7 was selected because it corresponded to the median NLR in the present cohort and falls within the range of cut-offs reported in prior ACS/AMI studies [12-14].

Investigations

All study subjects underwent a standardized set of investigations. These included a 12-lead ECG, complete blood count with differential counts, and biochemical investigations such as random blood glucose, serum creatinine, serum triglycerides, total cholesterol, HDL, and LDL levels using an automated biochemistry analyzer (Erba Mannheim EM 200). Two-dimensional echocardiography was performed to assess cardiac function. Additionally, cardiac biomarkers were evaluated using a qualitative troponin assay (Roche TROP T Sensitive kit) to support the diagnosis of myocardial infarction.

Ethical considerations

Ethical approval for the study was obtained from the Institutional Review Board and Ethics Committee prior to commencement. The study was approved by the Institutional Ethics Committee (GSLMC/RC: 477-EC/477-09/18). Written informed consent was obtained from all participants before enrollment in the study. Confidentiality of patient information was strictly maintained throughout the study, and all procedures were conducted in accordance with ethical guidelines.

Statistical analysis

Data collected during the study were entered into Microsoft Excel 2010 and analyzed using SPSS version 20. Quantitative variables were expressed as mean ± SD, while qualitative variables were presented as frequencies and percentages. Statistical analysis was performed using the Chi-square test for comparison of categorical variables. The results were interpreted based on a significance level of p < 0.05, which was considered statistically significant.

## Results

The study included 103 participants, with the majority belonging to the age groups of 40-50 years and 50-60 years (28, 27.1% each). Males predominated (76, 73.7%) compared to females (27, 26.3%). Nearly half of the participants were smokers (48, 46.6%), while 44 (42.7%) reported alcohol consumption. These findings indicate a predominantly middle-aged male population with a high prevalence of lifestyle-related risk factors (Table 1).

**Table 1:** Baseline demographic and risk factor profile of the study participants (n = 103).

Variable	Category	n	%
Age group (years)	<40	11	10.6
	40-50	28	27.1
	50-60	28	27.1
	60-70	21	20.4
	>70	15	14.6
Gender	Male	76	73.7
	Female	27	26.3
Smoking	Yes	48	46.6
	No	55	53.4
Alcohol consumption	Yes	44	42.7
	No	59	57.3

Table 2 shows that among the participants, hypertension was present in 42 (40.7%) and diabetes mellitus in 57 (55.4%), indicating a high burden of metabolic risk factors. The majority of cases were STEMI (92, 89.4%), while UA and NSTEMI were relatively less frequent. Hyperlipidemia was observed in 44 (42.7%) patients. Renal dysfunction was present in 43 (41.7%), and LV dysfunction was noted in 63 (61.2%) patients. Heart failure occurred in 54 (52.4%), and in-hospital mortality was recorded in 27 (26.2%) patients.

**Table 2:** Clinical profile and outcomes among study participants (n = 103). LV: left ventricular; NSTEMI: non-ST-elevation myocardial infarction; STEMI: ST-elevation myocardial infarction.

Variable	Category	n	%
Hypertension	Yes	42	40.7
	No	61	59.3
Diabetes mellitus	Yes	57	55.3
	No	46	44.7
Type of ACS	Stable/Unstable angina	10	9.7
	NSTEMI	1	1
	STEMI	92	89.3
Hyperlipidemia	Yes	44	42.7
	No	59	57.3
Serum creatinine	Normal (<1.2 mg/dL)	60	58.3
	Abnormal (≥1.2 mg/dL)	43	41.7
LV dysfunction (EF <50%)	Yes	63	61.2
	No	40	38.8
Arrhythmias	Present	36	35
	Absent	67	65
Complications	Present	83	80.6
	Absent	20	19.4
Myocardial infarction	Present	93	90.3
	Absent	10	9.7
Heart failure	Yes	54	52.4
	No	49	47.6
Mortality	Yes	27	26.2
	No	76	73.8

**Figure 1:**
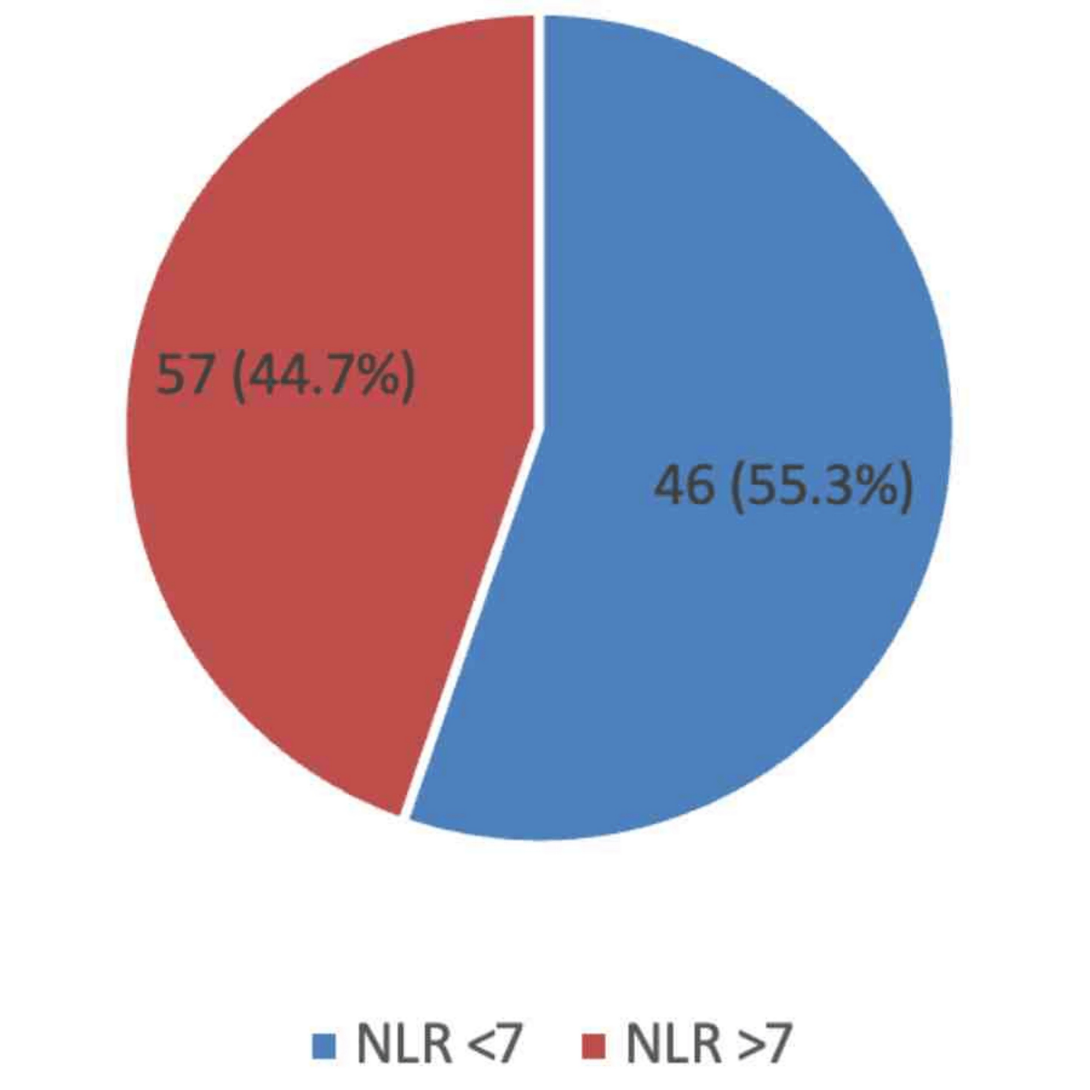
Distribution of neutrophil-to-lymphocyte ratio among the study participants (n = 103). NLR: Neutrophil-to-lymphocyte ratio.

Figure 1 shows that among the 103 study participants, 57 (55.3%) had an NLR value of less than 7, while 46 (44.7%) had an elevated NLR of 7 or more. The mean NLR was 9.2 ± 7.80, and the median NLR was 7 (range: 1-34). The distribution of NLR was nearly equal between the two groups, with a slight predominance of patients having lower NLR values.

Table 3 shows the site of infarction based on ECG findings. Inferior wall myocardial infarction was the most common presentation, accounting for 35 cases (37.6%), followed by anterior wall MI in 21 patients (22.6%) and anterolateral MI in 15 (16.1%). Anteroseptal MI was observed in 9 (9.7%) patients, while extensive MI accounted for 6 (6.5%) cases. Less frequent infarct locations included anteroinferior MI (2, 2.2%) and rare patterns such as inferoposterior MI (2, 2.2%), inferoseptal MI (1, 1.1%), inferolateral MI (1, 1.1%), and lateral wall MI (1, 1.1%).

**Table 3:** Site of infarction based on ECG findings (n = 93). MI: Myocardial infarction.

Site of infarct	n	%
Inferior wall MI	35	37.6
Anterior wall MI	21	22.6
Anterolateral MI	15	16.1
Anteroseptal MI	9	9.7
Extensive MI	6	6.5
Anteroinferior MI	2	2.2
Inferoposterior MI	2	2.2
Inferoseptal MI	1	1.1
Inferolateral MI	1	1.1
Lateral wall MI	1	1.1

Table 4 shows the association of demographic and clinical risk factors with NLR among the study participants. Age ≥50 years was significantly associated with elevated NLR, with 32 (54.2%) patients showing NLR ≥7 compared to 14 (31.8%) among younger patients (χ² = 6.12, p = 0.013). Smoking and alcohol consumption were also significantly associated with higher NLR, observed in 28 (58.3%) smokers (p = 0.028) and 25 (56.8%) alcohol users (p = 0.046). Hypertension and diabetes mellitus demonstrated significant associations with elevated NLR, with 24 (57.1%) hypertensive patients and 31 (54.4%) diabetic patients having NLR ≥7. Gender did not show a statistically significant association (p = 0.396). Renal dysfunction showed the strongest association, with 26 (60.5%) patients with abnormal creatinine having elevated NLR (χ² = 9.84, p = 0.002), indicating a strong link between inflammation and organ dysfunction.

**Table 4:** Association of demographic and clinical risk factors with NLR among study participants (n = 103). Test used: Chi-square test *p < 0.05 was considered statistically significant. NLR: Neutrophil-to-lymphocyte ratio.

Variable	Category	NLR <7 n (%) (57)	NLR ≥7 n (%) (46)	Total	χ²	p-value
Age group (years)	<50	30 (68.2)	14 (31.8)	44	6.12	0.013*
	≥50	27 (45.8)	32 (54.2)	59		
Gender	Male	38 (50.0)	38 (50.0)	76	0.72	0.396
	Female	19 (70.4)	8 (29.6)	27		
Smoking	Yes	20 (41.7)	28 (58.3)	48	4.85	0.028*
	No	37 (67.3)	18 (32.7)	55		
Alcohol consumption	Yes	19 (43.2)	25 (56.8)	44	3.98	0.046*
	No	38 (64.4)	21 (35.6)	59		
Hypertension	Yes	18 (42.9)	24 (57.1)	42	4.12	0.042*
	No	39 (63.9)	22 (36.1)	61		
Diabetes mellitus	Yes	26 (45.6)	31 (54.4)	57	5.28	0.022*
	No	31 (67.4)	15 (32.6)	46		
Hyperlipidemia	Yes	20 (45.5)	24 (54.5)	44	3.65	0.056
	No	37 (62.7)	22 (37.3)	59		
Serum creatinine	Normal	40 (66.7)	20 (33.3)	60	9.84	0.002*
	Abnormal	17 (39.5)	26 (60.5)	43		

Table 5 shows the association of clinical outcomes with NLR among the study participants. Elevated NLR (≥7) showed a strong association with adverse clinical outcomes. LV dysfunction was present in 38 (60.3%) patients with high NLR compared to only 8 (20.0%) with low NLR (χ² = 11.42, p < 0.001). Similarly, heart failure was significantly more common among patients with elevated NLR (34, 63.0%) than among those with lower NLR (χ² = 12.88, p < 0.001). Arrhythmias were also more frequent in patients with high NLR (24, 66.7%) than in those with low NLR (χ² = 10.21, p = 0.001). Complications were significantly more common in patients with elevated NLR (43/46, 93.5%) than in those with lower NLR (40/57, 70.2%) (χ² = 8.83, p = 0.003). Ejection fraction demonstrated one of the strongest associations, with 38 (60.3%) patients with EF <50% showing elevated NLR compared to only 8 (20.0%) with normal EF (χ² = 14.92, p < 0.001), indicating a strong relationship between systemic inflammation and impaired cardiac function. Mortality showed the strongest association, with 21 (77.8%) deaths occurring in patients with NLR ≥7 (χ² = 16.54, p < 0.001). However, no significant association was observed between NLR and infarction status (p = 0.470).

**Table 5:** Association of clinical outcomes with NLR among study participants (n = 103). Test used: Chi-square test *p < 0.05 was considered statistically significant. NLR: Neutrophil-to-lymphocyte ratio.

Variable	Category	NLR <7 n (%) (57)	NLR ≥7 n (%) (46)	Total	χ²	p-value
LV dysfunction	Yes	25 (39.7)	38 (60.3)	63	11.42	<0.001*
	No	32 (80.0)	8 (20.0)	40		
Arrhythmias	Present	12 (33.3)	24 (66.7)	36	10.21	0.001*
	Absent	45 (67.2)	22 (32.8)	67		
Complications	Present	40 (70.2)	43 (93.5)	83	8.83	0.003*
	Absent	17 (29.8)	3 (6.5)	20		
Myocardial infarction	Present	50 (53.8)	43 (46.2)	93	0.52	0.47
	Absent	7 (70.0)	3 (30.0)	10		
Ejection fraction (EF)	≥50% (normal)	32 (80.0)	8 (20.0)	40	14.92	<0.001*
	<50% (LV dysfunction)	25 (39.7)	38 (60.3)	63		
Heart failure	Yes	20 (37.0)	34 (63.0)	54	12.88	<0.001*
	No	37 (75.5)	12 (24.5)	49		
Mortality	Yes	6 (22.2)	21 (77.8)	27	16.54	<0.001*
	No	51 (67.1)	25 (32.9)	76		

## Discussion

This cross-sectional observational study evaluated the role of NLR as a prognostic marker in patients presenting with ACS. A total of 103 patients were included and categorized into two groups based on an NLR cut-off value of 7, representing low (<7) and high (≥7) inflammatory states. The use of a cut-off value of 7 in the present study is consistent with previous studies, although variations exist in the literature, with reported thresholds ranging from 4.7 to 6.97 depending on population characteristics and study design [17, 18, 19]. Such variability may be attributed to geographic, ethnic, and methodological differences, as also suggested by Bain BJ [20].

Age ≥50 years was significantly associated with elevated NLR (p = 0.013). This finding is consistent with studies by Chen C et al. [21] and Men M et al. [22], which also reported no significant age-related variation in NLR. However, Gazi E et al. [23] observed a significant association, indicating that demographic variability may influence this relationship. Similarly, gender did not show a statistically significant association with NLR in the present study, suggesting that the inflammatory response measured by NLR may be independent of sex distribution in patients with ACS.

Hypertension was more prevalent in patients with high NLR compared to those with low NLR, and this difference was statistically significant (p = 0.042). This observation is consistent with findings from Ergelen M et al. [17] and Bajari R and Tak S [18], where no significant association was demonstrated. The lack of significance suggests that while hypertension contributes to atherosclerosis, it may not directly influence acute inflammatory markers such as NLR.

Diabetes mellitus was also more common among patients with elevated NLR, and the association was statistically significant (p = 0.022). Similar trends were observed in studies by Azab B et al. [19] and Muhmmed Suliman MA et al. [24], indicating that metabolic disorders may contribute to systemic inflammation but are not independently associated with NLR elevation. Dyslipidemia showed no significant association with NLR in the present study (p = 0.056), consistent with findings from Núñez J et al. [10] and Muhmmed Suliman MA et al. [24]. The variability across studies, including contrasting findings by Tamhane UU et al. [25], may be due to differences in lipid profiles, treatment status, and population characteristics.

A significant negative correlation was observed between NLR and ejection fraction (EF), indicating that higher NLR values were associated with poorer left ventricular function. Patients in the high-NLR group had lower mean EF compared to those in the low-NLR group, and this correlation was statistically significant (p < 0.001). These findings are consistent with the study by Duffy BK et al. [7], which demonstrated lower EF values in patients with elevated NLR. This suggests that systemic inflammation plays a critical role in myocardial dysfunction and adverse cardiac remodeling following ischemic injury.

Serum creatinine levels were higher in the high-NLR group compared to the low-NLR group, indicating an association between systemic inflammation and renal dysfunction. This finding aligns with studies by Ergelen M et al. [17], Tamhane UU et al. [25], and Gul U et al. [26], all of which reported higher creatinine levels in patients with elevated NLR.

The association between renal dysfunction and NLR may be explained by the interplay between inflammation, endothelial dysfunction, and reduced renal perfusion in ACS. A strong association was observed between elevated NLR and adverse clinical outcomes, including heart failure, arrhythmias, complications, and mortality. Heart failure was more common in the high-NLR group compared to the low-NLR group, with a statistically significant association (p < 0.001). Similar findings were reported by Punit Gupta P et al. [27], Bajari R and Tak S [18], and Grzybowski M et al. [28], indicating that elevated NLR is a marker of poor cardiac function and increased hemodynamic compromise.

Arrhythmias were also more frequently observed in patients with high NLR, and the association was statistically significant (p = 0.001). This finding is consistent with studies reporting a significant association, such as Gul U et al. [26]. The variation may be due to differences in the types of arrhythmias and sample size. Complications were significantly higher in patients with elevated NLR (p = 0.003), with a larger proportion of high-NLR patients developing adverse events. This finding is consistent with studies by Gupta P et al. [27] and Gul U et al. [26], reinforcing the role of NLR as a predictor of in-hospital complications. Mortality showed a strong and statistically significant association with NLR, with higher death rates observed in patients with NLR ≥7 (p < 0.001). Similar trends were observed in studies by Gupta P et al. [27], Bajari R and Tak S [18], and Kaya MG et al. [29], confirming that elevated NLR is a reliable predictor of poor prognosis in patients with ACS.

Despite its strengths, the present study has a few limitations. First, the sample size was relatively small (n = 103), which may limit the generalizability of the findings. Second, the study was conducted at a single tertiary care center, which may introduce selection bias due to referral patterns. Third, the cross-sectional design limits the ability to establish causal relationships between NLR and clinical outcomes. Additionally, NLR was measured only at admission, and serial measurements were not performed, which could have provided better insight into dynamic inflammatory changes. The study also did not include multivariate analysis to adjust for confounding variables, which may affect the independent predictive value of NLR.

The findings of this study have important clinical implications. NLR is a simple, cost-effective, and readily available biomarker that can be derived from a routine complete blood count. Its strong association with adverse outcomes such as heart failure, complications, and mortality highlights its potential utility in early risk stratification of patients with ACS. In resource-limited settings, where advanced biomarkers may not be readily available, NLR can serve as a valuable tool for identifying high-risk patients who require closer monitoring and aggressive management. Incorporating NLR into clinical decision-making may improve patient outcomes by facilitating timely interventions. Future studies with larger sample sizes, multicentric designs, and longitudinal follow-up are required to validate these findings and establish standardized cut-off values for NLR across different populations. While this is a cross-sectional study, the use of a ROC curve in future studies could help identify the optimal threshold balancing sensitivity and specificity for mortality.

## Conclusions

The present study demonstrates that an elevated neutrophil-to-lymphocyte ratio (NLR ≥7) is significantly associated with adverse clinical outcomes in patients with ACS. Higher NLR values were associated with reduced EF, a higher incidence of complications, heart failure, arrhythmias, and increased in-hospital mortality. Traditional risk factors such as hypertension and diabetes mellitus showed statistically significant associations, whereas dyslipidemia did not. Overall, NLR emerged as a strong marker of systemic inflammation and disease severity. As a simple, cost-effective, and readily available parameter, NLR can be effectively used for early risk stratification and prognostication in patients with ACS, particularly in resource-limited settings. Incorporating NLR into routine clinical assessment may help identify high-risk patients and guide timely therapeutic interventions to improve outcomes.
